# Safety and feasibility of laparoscopic resection for giant hepatic hemangiomas: a retrospective cohort study

**DOI:** 10.1186/s12893-026-03580-1

**Published:** 2026-02-14

**Authors:** Aleksander Tarasik, Wiktoria Dzieżyk, Kamil Safiejko, Wojciech Fiedorowicz, Marcin Juchimiuk, Marian Domurat, Jerzy Hapanowicz, Hubert Puziuk, Mateusz Mucha, Tomasz Piotr Kozłowski, Anna Parfieniuk-Kowerda

**Affiliations:** 1 Department of Gastrointestinal Tumors, Maria Sklodowska-Curie Bialystok Oncology Center, Ogrodowa 12, Bialystok, 15-027 Poland; 2 Department of Infectious Diseases and Hepatology, Medical University of Bialystok, M. Sklodowskiej-Curie 24A, Bialystok, 15-276 Poland

**Keywords:** Hepatectomy, Cavernous hemangioma

## Abstract

**Background:**

Giant hepatic hemangiomas can cause significant symptoms or complications, and laparoscopic resection remains technically demanding. This study assessed the safety and feasibility of laparoscopic resection for giant hemangiomas.

**Methods:**

A retrospective review was conducted of all patients undergoing laparoscopic resection (LR) for giant hepatic hemangiomas between July 2021 and April 2025 at a single tertiary center. Surgical indication was based on the presence of clinically relevant symptoms reasonably attributable to the hemangioma, after exclusion of alternative causes.In selected symptomatic patients, documented tumor growth (>1 cm/year) and unfavorable anatomical location, particularly proximity to major hepatic vessels, were considered supportive factors rather than independent indications for surgery.Demographic, operative, and postoperative data were analyzed. Outcomes were compared by tumor location (left vs right hepatic lobe) using the Mann–Whitney U test, with effect sizes expressed as Hodges–Lehmann median differences.

**Results:**

Eighteen patients (83% female; median age 52 years) underwent laparoscopic resection with no conversions to open surgery. Median tumor size was 100 [IQR 90–140]. Median operative time was 227.5 [IQR 181.25–262.5], median blood loss 400 [IQR 325–575] and hospital stay 4.5 [IQR 4.0–5.0]. One minor postoperative complication classified as Clavien–Dindo grade II was observed. There were no major complications and no 90-day mortality. Operative time was significantly longer for right-lobe tumors (median 238 min [IQR 217–298]) vs left-lobe tumors (173 min [IQR 156–215]; Hodges–Lehmann difference 70 min, 95% CI 0–140; p = 0.049). Blood loss was higher in the right-lobe group (median 450 vs 350 ml), but this did not reach significance (p = 0.20). No recurrences were observed during follow-up (median 10 months).

**Conclusions:**

Laparoscopic resection of giant hepatic hemangiomas appeared to be feasible and safe in selected patients in this single-center experience. Right-lobe tumors were associated with longer operative times, reflecting greater technical complexity.

## Background

Hepatic hemangiomas are benign liver tumors with an estimated prevalence of approximately 2–3%. The vast majority of hemangiomas are small, remain stable throughout a patient’s life, exhibit minimal growth, and do not cause symptoms. This explains the difficulty in precisely determining their incidence. These lesions are typically clinically relevant only in the context of differential diagnosis with other hepatic tumors. Nevertheless, according to the literature, approximately 0.2–1% of patients develop giant hepatic hemangiomas [1].

The definition of a giant hepatic hemangioma remains inconsistent and varies among sources. Some authors define lesions larger than 5 cm as “giant” [2], whereas others reserve the term for lesions exceeding 10 cm [3]. Hemangiomas larger than 20 cm are now referred to in the literature as “very giant hemangiomas” [4]. This distinction is clinically important, as only hemangiomas exceeding 5 cm in diameter are considered to have clinical significance. In the present study, the term ‘giant hepatic hemangioma’ was used for descriptive purposes to denote lesions ≥ 10 cm in maximal diameter, acknowledging that no universally accepted size-based definition currently exists.

In patients with giant hemangiomas, the most common symptom is upper abdominal or right-sided pain, typically described as a dull ache that worsens with changes in body position and may resemble a sensation of a “foreign body” in the abdomen. Symptoms resulting from compression of adjacent organs may also occur, including nausea, dysphagia, or unintentional weight loss [5]. Less commonly, giant hemangiomas may cause signs of portal hypertension or jaundice due to compression of the Glisson’s sheath. Rare complications reported in the literature include thrombosis, hemobilia, cirrhosis due to multiple hemangiomas and chronic hepatic compression, and Kasabach–Merritt syndrome [6, 7]. The latter refers to vascular lesions associated with thrombocytopenia caused by platelet trapping and activation, followed by consumptive coagulopathy and purpura. One of the most severe complications is spontaneous rupture of a giant hemangioma, which may result in massive hemorrhage and hemorrhagic shock. Although extremely rare, this complication carries a mortality rate ranging from 60% to 83%. It is important to note that a significant proportion of patients with giant hemangiomas remain asymptomatic.

The oncologic relevance of giant hemangiomas primarily concerns their differentiation from other focal hepatic lesions, both benign and malignant. Advances in imaging techniques have enabled accurate diagnosis in most cases, allowing for non-invasive confirmation of giant hemangioma. However, occasionally ambiguous cases may require histological verification via biopsy. The literature contains no confirmed reports of malignant transformation of giant hemangiomas, which supports their classification as benign and oncologically stable. Nonetheless, diagnostic challenges may arise due to radiological similarities to rare malignant vascular tumors such as epithelioid hemangioendothelioma (EHE) or angiosarcoma. In particular, EHE, an intermediate-grade malignancy, may mimic hemangioma both clinically and radiologically in its early stages, potentially delaying diagnosis. In cases of atypical imaging findings, lesion progression, or systemic symptoms, an extended diagnostic workup should be considered, and these entities should be included in the differential diagnosis [8, 9].

All of the above highlights the importance of accurate diagnosis, appropriate monitoring, and—when indicated—adequate treatment of patients with giant hemangiomas. Currently, the treatment of choice for symptomatic giant hepatic hemangiomas is surgical removal of the lesion, particularly in cases of pain, rapid growth, or risk of serious complications such as hemorrhage [10, 11]. Surgical strategies include enucleation or partial hepatectomy. However, no consensus exists regarding the optimal surgical approach. Despite a growing number of reports suggesting advantages of laparoscopic techniques, most resections are still performed via open surgery. For example, in the study by Liu et al., 61% of patients underwent open procedures, while laparoscopic resection was associated with significantly lower blood loss, a reduced complication rate (4.1% vs. 19.2%), and shorter hospital stay [12]. Similar results were reported in a meta-analysis by Jien and Xiaohua involving 1,367 patients, in which open resections accounted for 54.6% of all procedures. This study also demonstrated the advantages of laparoscopy in terms of intraoperative blood loss, hospital stay, postoperative pain, and complication rates [13]. Similar favorable perioperative outcomes have also been achieved in our previous institutional series on laparoscopic liver resections for benign and malignant tumors [14], and national data confirm a steady increase in the adoption of laparoscopic liver surgery across Polish centers [15].

Given the limited number of publications and the relevance of this topic, we present our single-center experience with laparoscopic resection of symptomatic giant hepatic hemangiomas.

## Methods

### Study design and setting

This was a single-center retrospective study conducted at the Department of Gastrointestinal Surgery, Białystok Oncology Center (Poland). The study included all patients who underwent laparoscopic resection of giant hepatic hemangiomas between July 2021 and April 2025. During the study period, all eligible patients were treated using a laparoscopic approach; no open resections were performed.

### Patient selection

Patients were considered eligible for surgical treatment when giant hepatic hemangiomas were associated with clinically relevant symptoms reasonably attributable to the lesion, after exclusion of alternative causes of abdominal pain based on imaging and endoscopic evaluation. Symptom status at the time of surgical qualification was determined through routine clinical assessment and patient-reported complaints documented in the medical records.

In selected symptomatic patients, documented tumor growth during follow-up and unfavorable anatomical location, particularly proximity to major hepatic vessels, were considered as supportive factors during multidisciplinary decision-making, rather than isolated indications for surgery. The indication for surgical treatment was established following thorough discussion with the patient, including realistic expectations regarding symptom resolution, potential risks, and alternative management strategies. Hemangioma size alone was not considered an indication for surgical treatment.

Importantly, inclusion in the study was not restricted by a strict size threshold; patients with symptomatic hemangiomas slightly below the commonly used ‘giant’ cut-off were included when clinically relevant symptoms and documented tumor growth justified surgical treatment.

### Preoperative evaluation

Diagnosis was established using contrast-enhanced computed tomography (CT) and/or magnetic resonance imaging (MRI). In selected cases, ultrasound (US) was additionally used to monitor tumor progression over time. Upper and lower gastrointestinal endoscopic evaluations were performed as part of the preoperative workup to exclude alternative etiologies of abdominal symptoms.

In diagnostically inconclusive cases, ultrasound-guided core needle biopsy was performed. The biopsy tract was carefully planned to pass through normal liver parenchyma before reaching the lesion capsule, in order to minimize the risk of bleeding. No biopsy-related complications were observed.

### Surgical technique

**Fig. 1:**
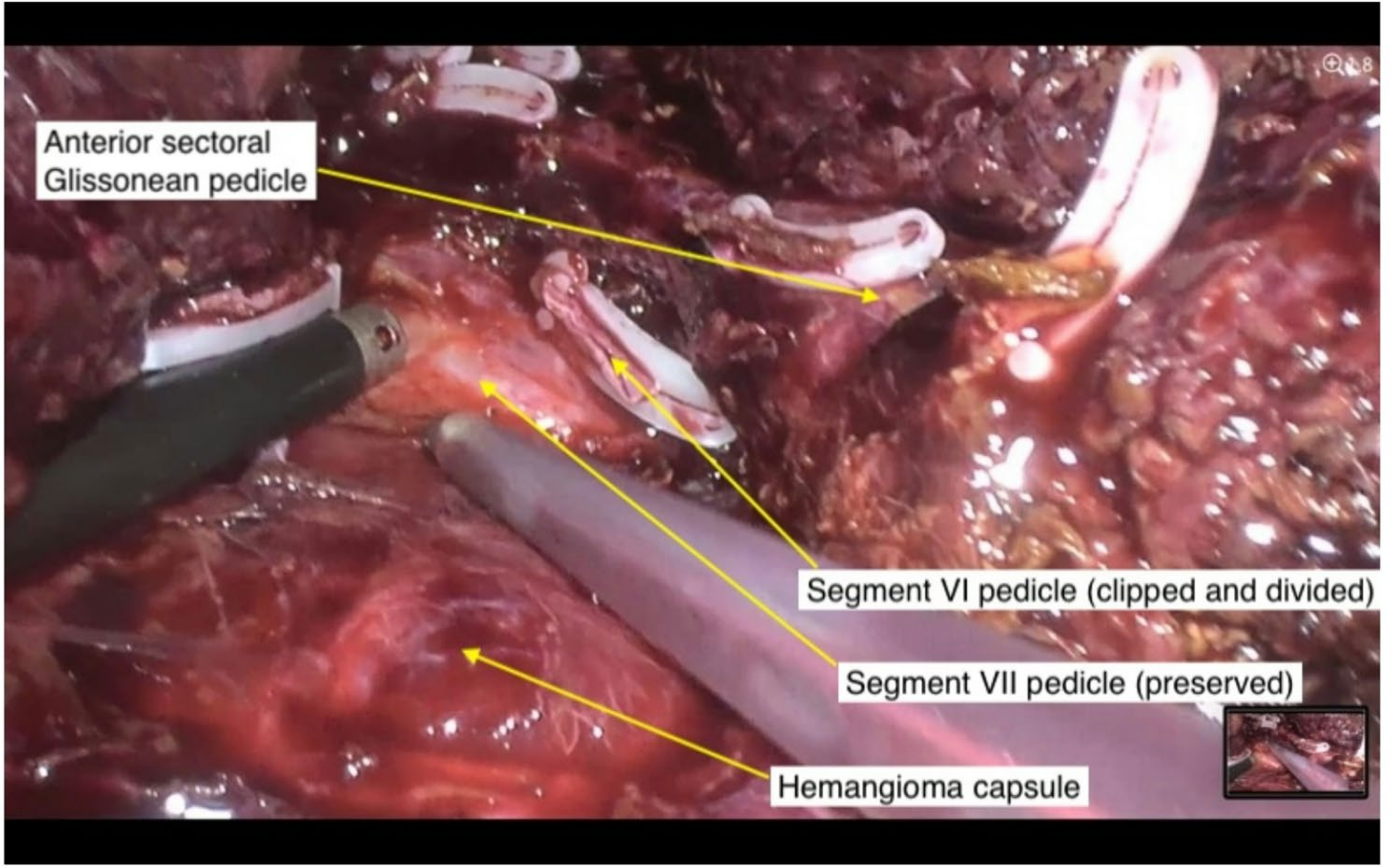
Dissection of the posterior right Glissonean pedicle. Using CUSA, the pedicle to segment VII (G7) is dissected from the hemangioma capsule. Clips were used to divide branches to anterior segments and segment VI

All procedures were performed laparoscopically. Intraoperative ultrasound (IOUS) was routinely used to assess tumor-vessel relationships and define resection planes. Whenever possible, parenchymal-sparing techniques were employed. In many cases, this required extensive dissection of segmental Glissonean pedicles to avoid major hepatectomies and preserve uninvolved liver tissue (Fig. 1).

**Fig. 2:**
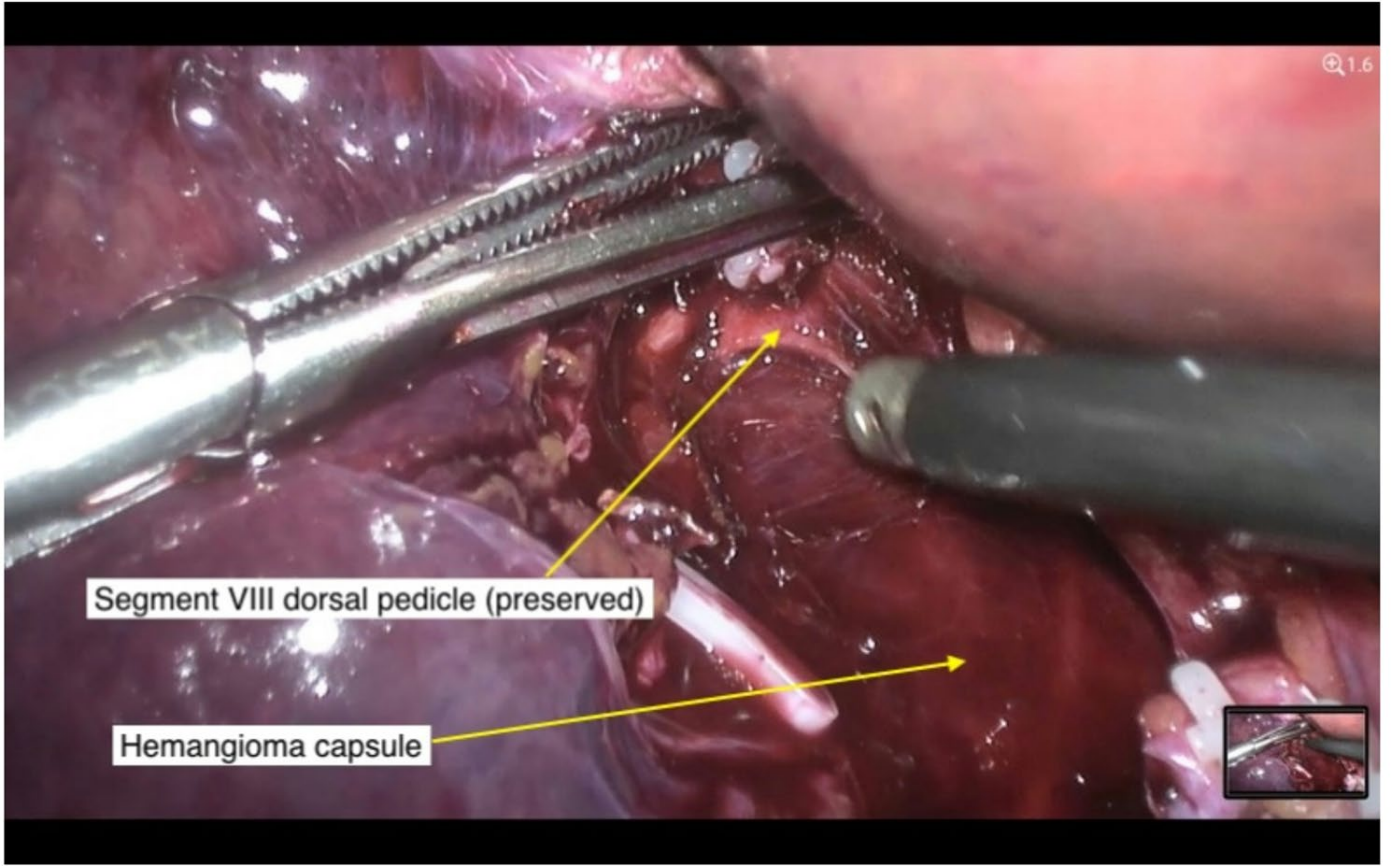
Dissection of segment VIII pedicle from hemangioma capsule.Dissection of distal Glissonean pedicle to segment VIII (G8d) directly from the hemangioma capsule, prior to stapler application

Resection was typically initiated by isolating the segmental Glissonean pedicle supplying the hemangioma, as identified during IOUS mapping. Selective control of this pedicle allowed for a parenchymal-sparing approach and facilitated bloodless dissection along the resection plane (Fig. 2).

**Fig. 3:**
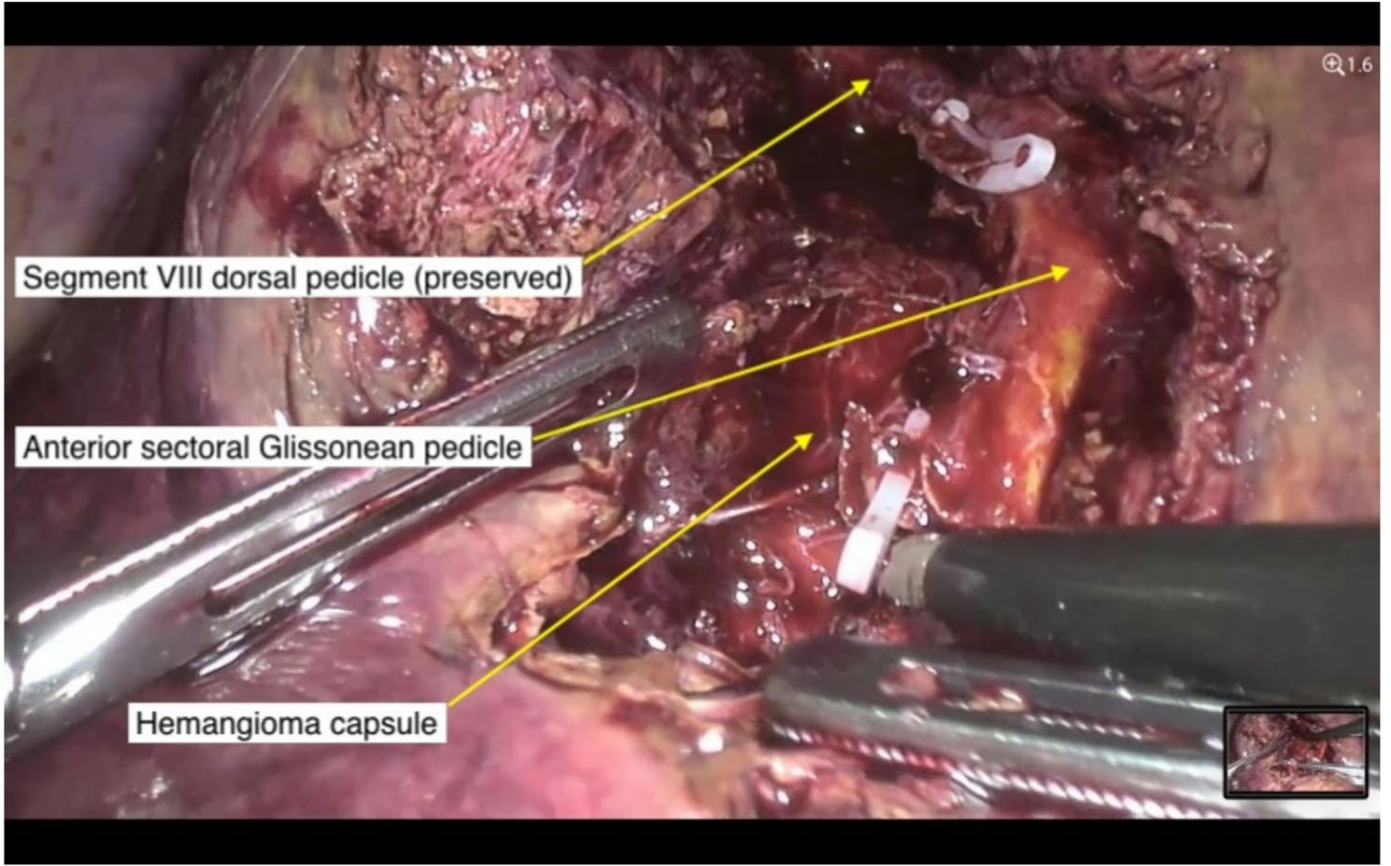
Anterior Glissonean pedicle exposed via wide hepatotomy.A laparoscopic view demonstrating the anatomical course of the anterior Glissonean pedicle after wide hepatotomy. The dissection clearly delineates the pedicle’s path and its interface with the adjacent hemangioma

For lesions in lateral or posterior segments, non-anatomical resections were preferred, maintaining a 0.5 cm margin from the lesion capsule. For centrally located lesions, enucleation was the preferred strategy when feasible (Fig. 3). In cases where tumors were adherent to major hepatic veins, inter-Laennec dissection was used to separate the tumor along the natural plane between the hepatic and cardiac Laennec’s capsules. All resections were prepared for potential use of the Pringle maneuver. Intermittent inflow occlusion was applied selectively when deemed necessary. Specimens were extracted via a Pfannenstiel minilaparotomy.

### Data collection and outcomes

The following variables were collected: patient age, sex, body mass index (BMI), comorbidities, tumor size and location (segmental classification), operative time, intraoperative blood loss, length of hospital stay, and postoperative complications. Concomitant procedures such as cholecystectomy were recorded. Multiple hemangiomas were present in some patients; surgical treatment was directed toward the dominant lesion selected for resection based on clinical and anatomical considerations. Postoperative complications were retrospectively identified through review of electronic medical records and follow-up outpatient visits and classified according to the Clavien–Dindo classification. Postoperative follow-up was based on outpatient clinic visits and imaging studies. Patients were evaluated for late postoperative complications. Follow-up imaging consisted of abdominal ultrasound, with additional cross-sectional imaging performed when clinically indicated.

### Statistical analysis

Data were analyzed descriptively. Continuous variables were reported as medians with interquartile ranges (IQR). Differences in operative parameters by tumor location (left vs. right hepatic lobe) were compared using the two-sided Mann–Whitney U test. Effect sizes were expressed as Hodges–Lehmann median differences with 95% confidence intervals (CI). A p-value < 0.05 was considered statistically significant.

## Results

The study group included 15 women (83%) and 3 men (17%). All patients were symptomatic at the time of surgical qualification.

The median tumor diameter was 100 mm (IQR 90–140). Median age was 52 years (range 40–78 years), and median body mass index (BMI) was 27.6 kg/m² (range 21–35).

The most common comorbidity was arterial hypertension (33.3%), followed by isolated cases of chronic obstructive pulmonary disease, nephrolithiasis, gastroesophageal reflux disease, hyperlipidemia, gout, type 2 diabetes, and hyperparathyroidism. Multiple comorbidities were present in 22.2% of patients. All patients were classified as ASA II. No patient presented with Kasabach–Merritt syndrome or other giant hemangioma-related complications.

### Tumor location according to Couinaud liver segments is summarized in Table 1

Only 3 tumors (16.7%) were confined to a single segment, whereas 10 tumors (55.6%) involved two segments and 2 tumors (11.1%) spanned three adjacent segments. In 3 patients (16.7%), multiple hepatic hemangiomas were present; however, only the largest symptomatic lesion was resected.

**Table 1:** Patient-level tumor location by Couinaud liver segments

Patient No.	Involved Couinaud liver segments
1	S6, S7
2	S2, S3
3	S2, S3, S4
4	**S6**, S3 (multifocal)
5	S8
6	S5, S6
7	S6, S7
8	S6, S7, S8
9	S8, S4
10	S4
11	S6, S7
12	S2, S3
13	S7, S8
14	**S2**,** S4**, S8 (multifocal)
15	S6, S7
16	S7, S8
17	**S8**, S4 (multifocal)
18	S4

In cases with multifocal or extensive hemangiomas, more than one Couinaud segment could be involved. In cases of multifocal hemangiomas, the dominant segment containing the main tumor mass is printed in bold

According to the Brisbane 2000 classification, all procedures were classified as minor laparoscopic liver resections, reflecting the intentional use of parenchymal-sparing strategies despite large tumor size. Limited anatomical resections were combined with tumor enucleation whenever appropriate.

In 4 patients (22.2%), cholecystectomy was performed due to tumor proximity to the gallbladder. Intraoperative ultrasound was used in every case to assess tumor–vascular relations and resection planes. Perioperative outcomes are summarized in Table 2. One patient developed postoperative fever requiring antibiotic therapy (Clavien–Dindo grade II). No other intraoperative or postoperative complications were observed. Median length of hospital stay was 4.5 days (IQR 4.0–5.0), median estimated blood loss was 400 ml (IQR 325–575), and median operative time was 227.5 min (IQR 181.25–262.5). No patient required intraoperative or postoperative blood transfusion, and 90-day mortality was 0%.

**Table 2:** Perioperative outcomes (*n* = 18)

Parameter	Value
Median tumor size (mm)	100 (90–140)
Median operative time (min)	227.5 (181.25–262.5)
Median blood loss (ml)	400 (325–575)
Median length of hospital stay (days)	4.5 (4.0–5.0)
Clavien–Dindo grade II, n (%)	1 (5.6)
90-day mortality (%)	0 (0)

A subgroup analysis according to tumor location (left vs. right hepatic lobe) is presented in Table 3. In the comparative analysis of resections according to hemangioma location, right-lobe resections were performed more frequently than left-lobe resections (Table 3). Median intraoperative blood loss was 450 ml (IQR 400–613) for right-lobe lesions and 350 ml (IQR 225–475) for left-lobe lesions (Hodges–Lehmann difference 100 ml, 95% CI − 50 to 300 ml; *p* = 0.20, two-sided Mann–Whitney U test). Median intraoperative blood loss was numerically higher in right-lobe resections compared with left-lobe resections; however, this difference did not reach statistical significance (*p* = 0.20). No patient required intraoperative or postoperative blood transfusion.

Median operative time was significantly longer for right-lobe tumors (237.5 min (IQR 217.0–297.5)) compared with left-lobe tumors (172.5 min (IQR 156.25–215.0)) (Hodges–Lehmann difference 70 min, 95% CI 0–140 min; *p* = 0.049, two-sided Mann–Whitney U test).

Median length of hospital stay was 5.0 days (IQR 3.75–5.0) for right-lobe resections and 4.0 days (IQR 4.0–4.75) for left-lobe resections, with no statistically significant difference between groups.

The median follow-up duration was 10 months (range 4–49 months). During follow-up, no tumor recurrence or late postoperative complications were observed.

**Table 3:** Operative outcomes according to tumor location

Location	Number of cases	Blood loss (ml)	Operative time (min)	Length of hospital stay, days
Left lobe	6	350 (225–475)	172.5 (156.25–215.0)	4.0 ( 4.0–4.75)
Right lobe	12	450 (400–613)	237.5 (217.0–297.5)	5.0 (3.75–5.0)

Values are presented as median (interquartile range)

## Discussion

In this study, we evaluated the short-term outcomes of laparoscopic resection for giant symptomatic hepatic hemangiomas in a consecutive cohort of patients treated at a single high-volume hepatobiliary center. The procedures were completed laparoscopically in all cases, with one minor complication (Clavien–Dindo II) and no major morbidity, supporting the feasibility of a minimally invasive, parenchymal-sparing approach in selected patients. Importantly, all resections were classified as minor according to the Brisbane 2000 terminology, reflecting an intentional strategy aimed at preserving functional liver parenchyma, even in the presence of large lesions and proximity to major vascular structures.

Several retrospective series have reported favorable perioperative outcomes following laparoscopic resection of giant hepatic hemangiomas. Xie et al. described a cohort of patients undergoing laparoscopic resection with acceptable operative time, limited blood loss, and short hospital stay, without perioperative mortality or major complications. Similar findings were reported by Hu et al. and Yan et al., despite differences in tumor size, location, and sample size across studies [16–18]. Although direct comparisons are limited by heterogeneity in patient selection and reporting methods, the perioperative outcomes observed in our cohort are consistent with these previously published laparoscopic experiences, supporting the feasibility of minimally invasive management in selected patients.

Evidence from meta-analyses further supports the role of laparoscopy in the surgical management of hepatic hemangiomas. In a meta-analysis by Jien and Xiaohua, laparoscopic surgery was associated with favorable perioperative outcomes compared with open resection, including reduced blood loss, lower complication rates, and shorter hospital stay. Importantly, no differences were observed in terms of hemangioma recurrence [13]. While these findings suggest potential advantages of minimally invasive approaches, they should be interpreted cautiously, as the majority of included studies were retrospective and subject to selection bias. Consequently, the present study does not allow for direct comparison between laparoscopic and open surgery but rather contributes additional evidence regarding the safety and feasibility of a laparoscopic strategy.

In the present series, all patients were treated laparoscopically without conversion to open surgery, reflecting both careful patient selection and institutional experience in minimally invasive liver surgery. No open resections for giant hepatic hemangiomas were performed during the study period, which precluded any internal comparison between surgical approaches. Therefore, our findings should be interpreted as a single-center experience focused on laparoscopic management, providing additional evidence regarding its safety and feasibility in selected patients.

Overall, the available literature suggests the safety and feasibility of laparoscopic resection for hepatic hemangiomas in selected patients. However, given the retrospective nature of most published data and the lack of high-quality comparative studies, definitive conclusions regarding the superiority of laparoscopy over other surgical or interventional treatment modalities cannot be drawn. Further well-designed prospective studies are needed to better define the optimal management strategy for giant symptomatic hepatic hemangiomas.

Although all patients were symptomatic at the time of surgical qualification, postoperative symptom resolution was not systematically assessed in our cohort and therefore could not be reliably quantified. Data from multiple centers suggest potential clinical benefits of surgical treatment for symptomatic hepatic hemangiomas. In a large case series involving 204 patients, Aziz et al. reported symptom relief in the majority of patients following surgical resection. In a separate study, Miura et al. observed symptom resolution within 90 days postoperatively in 63% of patients [19, 20].

When comparing surgical strategies, multiple studies suggest that enucleation of hepatic hemangiomas yields better perioperative outcomes than formal hepatic resection. A large meta-analysis including 1,185 patients showed that both postoperative complication rates and intraoperative blood loss were significantly lower in the enucleation group [21].

Another study similarly confirmed the advantages of enucleation over standard hepatic resection in terms of perioperative outcomes. Enucleation was associated with shorter operative time, less blood loss, and a lower rate of postoperative complications. However, the number of blood transfusions and the duration of hepatic inflow occlusion (Pringle maneuver) were comparable between groups [22].

On the other hand, Zhang et al. (2024), in a comparative analysis of 127 resections and 287 enucleations for giant hepatic hemangiomas, reported that enucleation was associated with greater intraoperative blood loss compared with formal resection. In the same study, independent risk factors for increased blood loss included tumor size ≥ 10 cm, adjacency to major vessels, location in the right liver or caudate lobe, and a portal phase enhancement ratio ≥ 38.9%. Notably, the association between right-lobe location and higher bleeding risk is consistent with our findings, where right-sided resections were technically more demanding and often required more extensive vascular control [23].

It is possible that the use of a robotic approach may improve perioperative outcomes in the management of hepatic hemangiomas located in anatomically challenging areas. In a recent propensity score-matched study comparing robot-assisted and laparoscopic liver resection for large cavernous hemangiomas, perioperative outcomes were generally comparable between the two techniques. However, in cases where the tumor was adjacent to major vascular structures, the robotic approach was associated with reduced intraoperative blood loss. These findings suggest that robotic assistance may offer technical advantages in selected patients, although further studies are warranted to confirm these potential benefits [24].

Although surgical treatment remains the gold standard for managing giant symptomatic hepatic hemangiomas, nonsurgical interventional techniques have increasingly been explored as alternative treatment options in selected patients.

In their systematic review on transarterial embolization and chemoembolization (including transarterial lipiodolization using gelatin sponge, pingyangmycin, or bleomycin) in patients with hepatic hemangiomas, Furumaya et al. (2019) analyzed outcomes from 1,284 cases, 89.9% of which involved symptomatic lesions. The authors reported high efficacy: partial or complete symptom resolution was observed in 98.5% of patients. Tumor diameter was reduced in 89.9% of cases, from a mean of 9.79 cm to 4.00 ± 1.36 cm (*p* < 0.001) following embolization. The procedure also demonstrated good safety, with Clavien-Dindo grade 3 complications occurring in only 2.9% (37/1,284) of patients, and surgical intervention required in 2.7% (35/1,284). In some cases, multiple embolization sessions were required to achieve the desired therapeutic effect. No reduction in hemangioma size was observed in 89 patients (7.3%), while tumor enlargement occurred in 34 patients (2.8%). Follow-up data showed that complete symptom resolution was achieved in only 81 of 1,096 patients (7.4%), whereas partial improvement was reported in 999 patients (91.1%) [25].

In a recent review, Kacała et al. summarized available evidence suggesting that transarterial chemoembolization may result in a ≥ 50% reduction in hemangioma volume in a substantial proportion of patients, although post-embolization syndrome was relatively frequent [26].

A two-step treatment strategy combining transarterial embolization (TAE) with subsequent laparoscopic liver resection (LLR) has been explored to improve safety and technical feasibility in selected patients with giant hepatic hemangiomas. In a study by Della Corte et al. (2021), ten patients with eleven hemangiomas exceeding 10 cm underwent microparticle embolization of the hemangioma-feeding arteries, followed by LLR after a mean interval of 2.2 days. In all cases, resection was completed laparoscopically without conversion to open surgery, resulting in a 100% technical success rate. No perioperative complications or mortality were reported. These findings suggest that preoperative TAE may be a valuable adjunct to enhance the safety and efficacy of minimally invasive resection in complex cases [27].

Another minimally invasive interventional option for giant hepatic hemangiomas is percutaneous sclerotherapy. Ayoobi-Yazdi et al. (2021) evaluated this approach in 28 symptomatic patients using a combination of bleomycin and ethiodized oil. Complete symptom resolution at 12 months was achieved in 61% of patients, with partial improvement in the remaining cases. While the rate of complete relief was lower than that reported for surgical resection, the procedure demonstrated an excellent safety profile, with no major complications observed. Reported adverse events were mild and self-limiting, including transient pain, low-grade fever, and temporary elevation of liver enzymes. These results suggest that percutaneous sclerotherapy may be considered in selected patients who are not candidates for surgery or who prefer a less invasive approach [28].

Various ablative techniques have been described for the treatment of giant hepatic hemangiomas. In a meta-analysis, Fei et al. (2023) evaluated the effectiveness of microwave ablation in this setting. According to their findings, the mean lesion diameter decreased by 3.009 cm, while tumor volume was reduced by approximately 53.169%. The reported postoperative complication rate was very low at 1.7%, and no postoperative mortality was observed [29].

In a systematic review, Gao et al. evaluated the efficacy of radiofrequency ablation (RFA) for giant hepatic hemangiomas. The expert consensus from China cited several studies, highlighting a relatively high rate of complete ablation. In one of the referenced studies, in which the RFA technique was modified to reduce complication rates, complete ablation was achieved in 90% of patients. However, the complication rate remained high at 47.6%. Most adverse events were mild (Clavien-Dindo grade I), but other reports described serious complications, including rupture of the hemangioma during ablation, thermal injury to adjacent organs, massive hemolysis, diaphragmatic injury, acute respiratory distress syndrome (ARDS), and acute kidney injury. The authors suggested that performing RFA under laparoscopic guidance may help prevent some of these complications [30].

Given the limited high-quality data on the safety and efficacy of alternative approaches, surgical treatment remains the standard of care for giant symptomatic hepatic hemangiomas. The introduction of minimally invasive techniques has been associated with favorable perioperative outcomes and has contributed to improved safety of surgical management in selected patients. However, promising results reported for transarterial embolization—particularly its reported efficacy and acceptable complication rates—suggest that its use may increase in appropriately selected cases. Still, the relatively low rate of complete symptom resolution and the frequent need for repeated treatments highlight the need for further refinement and better patient selection. In cases of giant symptomatic hemangiomas located in anatomically challenging areas, preoperative embolization may be considered as an adjunct to reduce surgical complexity. In the future, tailored or combined treatment strategies may help optimize outcomes for patients with giant symptomatic hepatic hemangiomas.

### Limitations

Several limitations of this study should be acknowledged. This was a retrospective, single-center analysis, which may limit the generalizability of the results. However, the study was conducted in a high-volume hepatobiliary center performing approximately 70–80 laparoscopic liver resections annually, including around 10–15 procedures for benign liver tumors each year. All resections were performed or supervised by a single experienced senior surgeon, ensuring procedural consistency.

Although patients with benign liver tumors represent a larger referral population, only a selected subset of patients underwent surgical treatment based on multidisciplinary evaluation, which may introduce a degree of selection bias.

The lack of a universally accepted definition of ‘giant’ hepatic hemangioma may limit comparability between studies; therefore, we report detailed tumor size distributions rather than relying solely on terminology.

Another important limitation is that postoperative symptom resolution and clinical improvement were not systematically or prospectively assessed. In addition, the relatively short follow-up period and the relatively small sample size limit statistical power and preclude definitive conclusions regarding comparative effectiveness. These limitations are inherent to retrospective single-center studies of rare benign liver diseases and should be considered when interpreting the results.

## Conclusion

Laparoscopic resection of giant hepatic hemangiomas appears to be a safe and feasible treatment option in selected patients when performed in experienced centers. Right-lobe tumors are associated with longer operative times, reflecting greater technical complexity. Given the retrospective design and limited sample size, these findings should be interpreted with caution. Further prospective and comparative studies are needed to define the optimal role of laparoscopic resection in relation to other surgical approaches and alternative treatment strategies.

## Data Availability

The datasets generated and/or analyzed during the current study are not publicly available due to patient privacy regulations but are available from the corresponding author on reasonable request and with permission from the Białystok Oncology Center.

## Abbreviations

EHE: Epithelioid hemangioendothelioma
CT: Computed tomography
MRI: Magnetic resonance imaging
US: Ultrasound
IOUS: Intraoperative ultrasound
BMI: Body mass index
IQR: Interquartile range
SD: Standard deviation
CI: Confidence interval
COPD: Chronic obstructive pulmonary disease
GERD: Gastroesophageal reflux disease
ASA: American Society of Anesthesiologists
ALT: Alanine aminotransferase
AST: Aspartate aminotransferase
VAS: Visual analog scale
TACE: Transarterial chemoembolization
TAE: Transarterial embolization
LLR: Laparoscopic liver resection
RFA: Radiofrequency ablation
ARDS: Acute respiratory distress syndrome

## References

[1] Mocchegiani F, Vincenzi P, Coletta M, et al. Prevalence and clinical outcome of hepatic haemangioma with specific reference to the risk of rupture: A large retrospective cross-sectional study. Dig Liver Dis. 2016;48(3):309–14. 10.1016/j.dld.2015.09.016. 26514738 10.1016/j.dld.2015.09.016

[2] Leon M, Chavez L, Surani S. Hepatic hemangioma: what internists need to know. World J Gastroenterol. 2020;26(1):11–20. 10.3748/wjg.v26.i1.11. 31933511 10.3748/wjg.v26.i1.11 PMC6952297

[3] Di Carlo I, Koshy R, Al Mudares S, Ardiri A, Bertino G, Toro A. Giant cavernous liver hemangiomas: is it the time to change the size categories? Hepatobiliary Pancreat Dis Int. 2016;15(1):21–9. 10.1016/S1499-3872(15)60035-2. 26818540 10.1016/s1499-3872(15)60035-2

[4] Chang A, Ruch B, Khan A, Levy M, Sharma A. Giant liver hemangiomas: A plea for early surgical referral and resection. Case Rep Surg. 2020;2020:5923787. 10.1155/2020/5923787. Published 2020 Jun 16. 32607273 10.1155/2020/5923787 PMC7315262

[5] Kuo PC, Lewis WD, Jenkins RL. Treatment of giant hemangiomas of the liver by enucleation. J Am Coll Surg. 1994;178(1):49–53. 8156117

[6] Mikami T, Hirata K, Oikawa I, Kimura M, Kimura H. Hemobilia caused by a giant benign hemangioma of the liver: report of a case. Surg Today. 1998;28(9):948–52. 10.1007/s005950050259. 9744407 10.1007/s005950050259

[7] Pateron D, Babany G, Belghiti J, et al. Giant hemangioma of the liver with pain, fever, and abnormal liver tests. Report of two cases. Dig Dis Sci. 1991;36(4):524–7. 10.1007/BF01298887. 2007371 10.1007/BF01298887

[8] Bajenaru N, Balaban V, Săvulescu F, Campeanu I, Patrascu T. Hepatic hemangioma -review-. *J Med Life*. 2015;8 Spec Issue(Spec Issue):4–11. PMID: 25729512; PMCID: PMC4564031. PMC4564031 26361504

[9] Jang JK, Thomas R, Braschi-Amirfarzan M, Jagannathan JP. A review of the spectrum of imaging manifestations of epithelioid hemangioendothelioma. AJR Am J Roentgenol. 2020;215(5):1290–8. 10.2214/AJR.20.22876. 32841059 10.2214/AJR.20.22876

[10] Curry MP, Chopra S. Hepatic hemangioma. In: Lindor KD, Robson KM, eds. *UpToDate*. Waltham, MA: UpToDate Inc. Updated August 20, 2024. Accessed June 22, 2025 https://www.uptodate.com/contents/hepatic-hemangioma

[11] Gilon D, Slater PE, Benbassat J. Can decision analysis help in the management of giant hemangioma of the liver? J Clin Gastroenterol. 1991;13(3):255–8. 2066541

[12] Liu Q, Liu F, Ding J, Wei Y, Li B. Surgical outcomes and quality of life between laparoscopic and open approach for hepatic hemangioma: A propensity score matching analysis. Med (Baltim). 2019;98(6):e14485. 10.1097/MD.0000000000014485. 10.1097/MD.0000000000014485 PMC6380717 30732219

[13] Jien H, Xiaohua L. Laparoscopic versus open surgery in the treatment of hepatic hemangioma: A meta-analysis. Med (Baltim). 2021;100(8):e24155. 10.1097/MD.0000000000024155. 10.1097/MD.0000000000024155 PMC7909164 33663045

[14] Tarasik A, Łapiński TW. Laparoscopic resection for the treatment of liver tumors. Clin Oncol. 2021;6:1813. Available from: https://www.clinicsinoncology.com/open-access/laparoscopic-resection-for-the-treatment-of-liver-tumors-6923.pdf

[15] Hołówko W, Serednicki W, Bartkowiak M, Wysocki M, Domurat M, Mielko J, Pierściński S, Hogendorf P, Masior Ł, Kalinowski P, Wierdak M, Frączek M, Tarasik A, Wróblewski T, Budzyński A, Pędziwiatr M, Grąt M. Early adoption of laparoscopic liver surgery in poland: a National retrospective cohort study. Int J Surg. 2024;110:361–71. 10.1097/JS9.0000000000000840. 37816169 10.1097/JS9.0000000000000840 PMC10793755

[16] Xie QS, Chen ZX, Zhao YJ, Gu H, Geng XP, Liu FB. Outcomes of surgery for giant hepatic hemangioma. BMC Surg. 2021;21(1):186. 10.1186/s12893-021-01185-4. Published 2021 Apr 8. 33832476 10.1186/s12893-021-01185-4 PMC8033692

[17] Yan C, Li BH, Sun XT, Yu DC. Laparoscopic hepatectomy is superior to open procedures for hepatic hemangioma. Hepatobiliary Pancreat Dis Int. 2021;20(2):142–6. 10.1016/j.hbpd.2020.09.001. 32980268 10.1016/j.hbpd.2020.09.001

[18] Hu M, Chen K, Zhang X, Li C, Song D, Liu R. Robotic, laparoscopic or open hemihepatectomy for giant liver haemangiomas over 10 cm in diameter. BMC Surg. 2020;20(1):93. 10.1186/s12893-020-00760-5. 32375738 10.1186/s12893-020-00760-5 PMC7204244

[19] Miura JT, Amini A, Schmocker R, et al. Surgical management of hepatic hemangiomas: a multi-institutional experience. HPB (Oxford). 2014;16(10):924–8. 10.1111/hpb.12291. 24946109 10.1111/hpb.12291 PMC4238859

[20] Aziz H, Brown ZJ, Baghdadi A, Kamel IR, Pawlik TM. A comprehensive review of hepatic hemangioma management. J Gastrointest Surg. 2022;26(9):1998–2007. 10.1007/s11605-022-05382-1. 35705835 10.1007/s11605-022-05382-1

[21] Liu Y, Wei X, Wang K, et al. Enucleation versus anatomic resection for giant hepatic hemangioma: A Meta-Analysis. Gastrointest Tumors. 2017;3(3–4):153–62. 10.1159/000455846. 28611982 10.1159/000455846 PMC5465724

[22] Jiang B, Shen ZC, Fang XS, Wang XM. Enucleation versus hepatectomy for hepatic hemangiomas: A meta-analysis. Front Surg. 2022;9:960768. 10.3389/fsurg.2022.960768. Published 2022 Jul 28. 35965862 10.3389/fsurg.2022.960768 PMC9366102

[23] Zhang H, Xu H, Wen N, Li B, Chen K, Wei Y. Laparoscopic liver resection or enucleation for giant hepatic hemangioma: how to choose? Surg Endosc. 2024;38(6):3079–87. 10.1007/s00464-024-10820-z. 38622227 10.1007/s00464-024-10820-z

[24] Zhang W, Liu J, Zhang Z, et al. Perioperative outcomes of robot-assisted versus laparoscopic liver resection for cavernous hemangioma: a propensity score matching study. Surg Endosc. 2023;37(6):4505–16. 10.1007/s00464-022-09834-2. 36810688 10.1007/s00464-022-09834-2 PMC10234931

[25] Furumaya A, van Rosmalen BV, Takkenberg RB, et al. Transarterial (Chemo-)Embolization and lipiodolization for hepatic haemangioma. Cardiovasc Intervent Radiol. 2019;42(6):800–11. 10.1007/s00270-019-02169-x. 30783780 10.1007/s00270-019-02169-x PMC6503075

[26] Kacała A, Dorochowicz M, Matus I, et al. Hepatic hemangioma: review of imaging and therapeutic strategies. Med (Kaunas). 2024;60(3):449. 10.3390/medicina60030449. 10.3390/medicina60030449 PMC10972168 38541175

[27] Della Corte A, Marino R, Ratti F, et al. The Two-Step treatment for giant hepatic hemangiomas. J Clin Med. 2021;10(19):4381. 10.3390/jcm10194381. Published 2021 Sep 25. 34640399 10.3390/jcm10194381 PMC8509141

[28] Ayoobi-Yazdi N, Mehrabinejad MM, Dashti H et al. Percutaneous sclerotherapy with bleomycin and ethiodized oil: A promising treatment in giant hepatic hemangioma. *Radiol*ogy. 2021;301(2):e444-e452. 10.1148/radiol.2021204444 10.1148/radiol.2021204444 34402664

[29] Fei L, Hongsong X. Effectiveness of microwave ablation for the treatment of hepatic hemangioma - meta-analysis and meta-regression. Int J Hyperth. 2023;40(1):2146214. 10.1080/02656736.2022.2146214. 10.1080/02656736.2022.2146214 36535918

[30] Gao J, Kong J, Ding XM, et al. Laparoscopic vs computerized tomography-guided radiofrequency ablation for large hepatic hemangiomas abutting the diaphragm. World J Gastroenterol. 2015;21(19):5941–9. 10.3748/wjg.v21.i19.5941. 26019459 10.3748/wjg.v21.i19.5941 PMC4438029

